# Hyperglycemia-induced downregulation of apolipoprotein M expression is not via the hexosamine pathway

**DOI:** 10.1186/s12944-015-0103-5

**Published:** 2015-09-16

**Authors:** Bo Jiang, Xiaoying Zhang, Dongmei Di, Guanghua Luo, Yuanping Shi, Jun Zhang, Maria Berggren-Söderlund, Peter Nilsson-Ehle, Ning Xu

**Affiliations:** Department of Cardiothoracic Surgery in the Third Affiliated Hospital, Soochow University, Changzhou, 213003 China; Comprehensive Laboratory, Third Affiliated Hospital, Soochow University, Changzhou, 213003 China; Division of Clinical Chemistry and Pharmacology, Department of Laboratory Medicine, Lund University, S-221 85 Lund, Sweden

**Keywords:** Apolipoprotein M, Glucosamine, Hyperglycemia, Hexosamine pathway

## Abstract

**Background:**

We previously demonstrated that hyperglycemia could suppress apolipoprotein M (apoM) synthesis both *in vivo* and *in vitro*; however, the mechanism of hyperglycemia-induced downregulation of apoM expression is unknown yet.

**Methods:**

In the present study we further examined if hexosamine pathway, one of the most important pathways of glucose turnover, being involved in modulating apoM expression in the hyperglycemia condition. We examined the effect of glucosamine, a prominent component of hexosamine pathway and intracellular mediator of insulin resistance, on apoM expression in HepG2 cells and in rat’s models. In the present study we also determined apolipoprotein A1 (apoA1) as a control gene.

**Results:**

Our results demonstrated that glucosamine could even up-regulate both apoM and apoA1 expressions in HepG2 cell cultures. The glucosamine induced upregulation of apoM expression could be blocked by addition of azaserine, an inhibitor of hexosamine pathway. Moreover, intravenous infusion of glucosamine could enhance hepatic apoM expression in rats, although serum apoM levels were not significantly influences.

**Conclusions:**

It is concluded that both exogenous and endogenous glucosamine were essential for the over-expression of apoM, which may suggest that the increased intracellular content of glucosamine does not be responsible for the depressed apoM expression at hyperglycemia condition.

## Introduction

Apolipoprotein M, discovered by Ning Xu and Dahlbäck in 1999, is mainly located in high-density lipoprotein (HDL) in the blood [1]. By influencing preβ-HDL formation and cholesterol efflux, apoM is thought to be an important regulative factor in HDL metabolism and further affects on the initiation and development of atherosclerosis [2]. ApoM, *in vivo*, could be function as an acceptor of sphingosine- 1-phosphate and could enhance HDL-mediated anti-oxidation effects [3, 4]. It has been suggested that apoM may associate with coronary heart disease (CHD), diabetes and other diseases with dyslipidemia [5]. The serum apoM concentration is significantly reduced in diabetic patients [6]. Single nucleotide polymorphism (SNP) T-778C, C-724del and T-855C in the proximal promoter region of apoM gene have significant associations with CAD and type-2 diabetes among Han Chinese [7, 8]. Moreover, apoM is reduced in diabetic rats and exogenous insulin administrations could partially reverse the abnormal apoM expression [9]. Intralipid could increase plasma FFA levels in rats, decrease insulin sensitivity and suppressed apoM expression [10]. Whereas in our cell culture experiments, insulin, insulin-like growth factor I (IGF-I) and IGF-I potential peptide (IGF-IPP) could significantly inhibit apoM expression in a dose- and a time-dependent manner [11]. The mechanism of this discrepancy of experimental result *in vivo* and *in vitro* is still unknown. We previously reported that hyperglycemia could significantly down-regulate apoM expression either *in vivo* or *in vitro* [12], which could be reversed by rosiglitazone *in vivo* [13], but it’s mechanism is unknown yet. Hyperglycemia could stimulate the hexosamine pathway and leads to the increase of the endogenous glucosamine and metabolites of glycosylation [14]. In the present we investigated if glucosamine, a common subject of hexosamine biosynthesis pathway,being responsible for hyperglycemia-induced suppression of apoM expression.

## Result

### Effects of glucosamine on the expressions of apoM and apoA1 in HepG2 cells

**Fig. 1:**
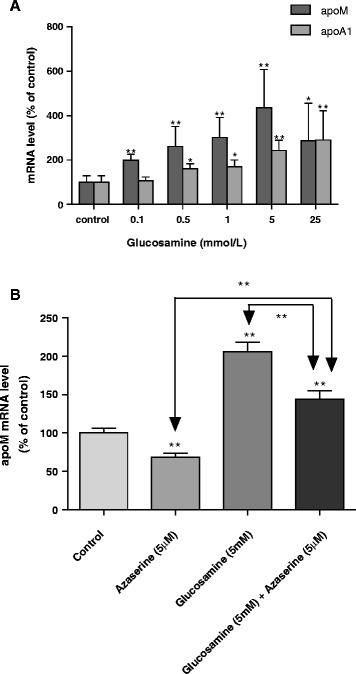
Effects of glucosamine on mRNA levels of apoM and ApoA1in HepG2 cells HepG2 cells were cultured with different concentrations of glucosamine (panel **a**) with or without azaserine (Panel **b**) for 24 h. In panel A, the mRNA levels of apoM and apoA1 were determined by real-time RT-PCR. In panel B, glucosamine was at 5 mmol/L and azaserine was at 5 μmol/L. The apoM mRNA levels were determined by real-time RT-PCR. Each experimental group contained 6 replicates and real-time RT-PCRs were run in triplicate. The control group is set 100 %. *P <0.05 and **P <0.01 vs. control group

In HepG2 cell cultures, the mRNA levels of apoM and apoA1 were significantly increased in the presence of glucosamine compared to the group without glucosamine (control group), with a dose-dependent manner (Fig. 1a). As shown in Fig. 1b, the cells cultured with azaserine alone, cells cultured with glucosamine alone and cells cultured with glucosamine together with azaserine, the apoM mRNA was decreased by about 32 % when cells cultured with azaserine alone compared to the control group (*P* < 0.01), which indicates that azaserine itself could inhibit apoM expression in HepG2 cells. The apoM mRNA levels were significantly increased when the cells cultured with glucosamine alone, whereas when the cells cultured with glucosamine in the presence of azaserine the apoM mRNA levels were much lower compared to the cells cultured with glucosamine alone.

### Effects of short-term glucosamine infusion on apoM synthesis and lipids profile in rats

**Fig. 2:**
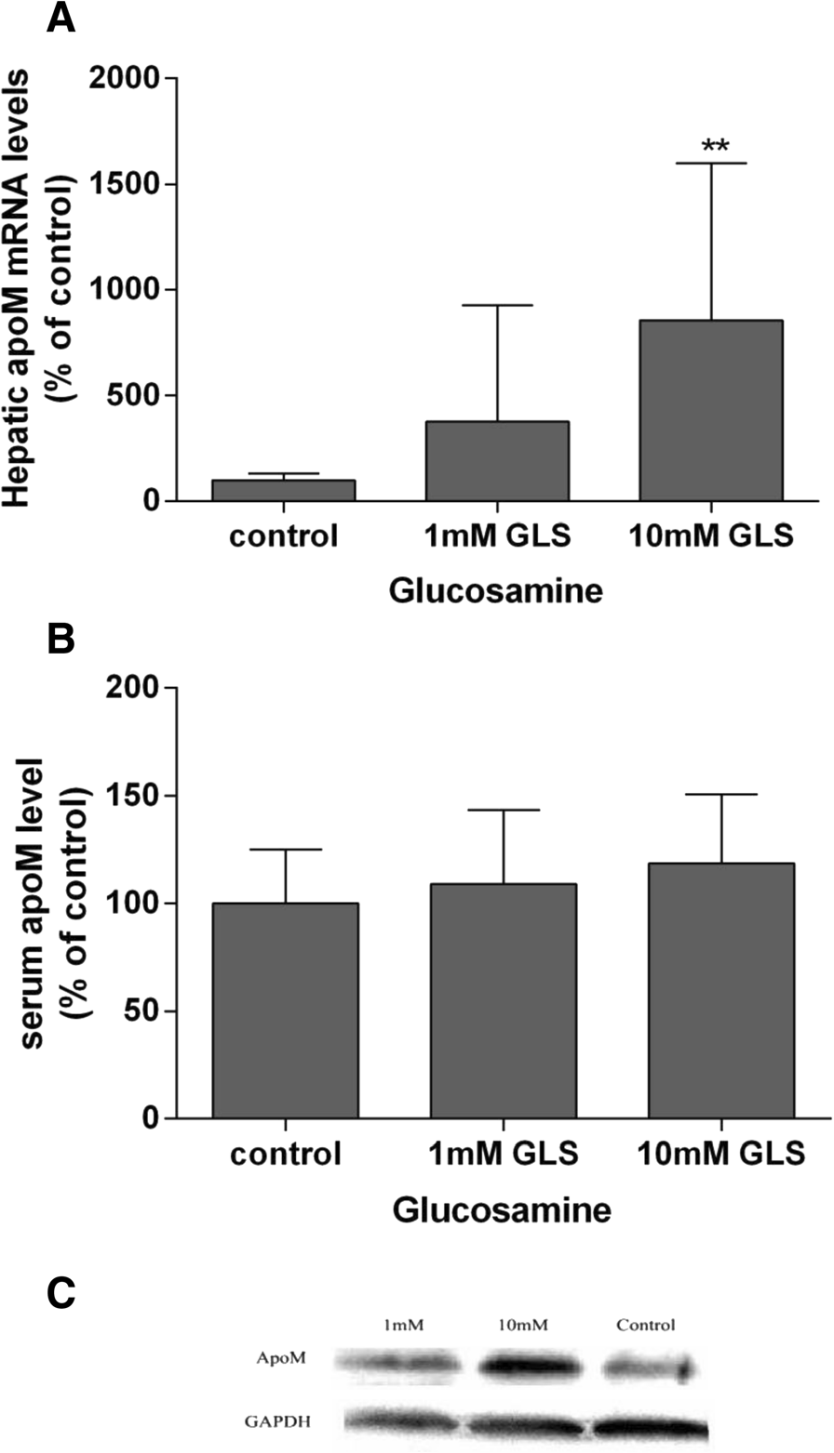
Effects of glucosamine on apoM synthesis in rats. Hepatic apoM mRNA levels (Panel **a**) were determined by real-time RT-PCR. Serum apoM levels (Panel **b**) were determined by Western-blot analysis as described in Materials and methods. A sample of Western-blot analysis is represented in panel **c**. The control group is set as 100 %. *P <0.05 and **P < 0.01 vs. control

**Fig. 3:**
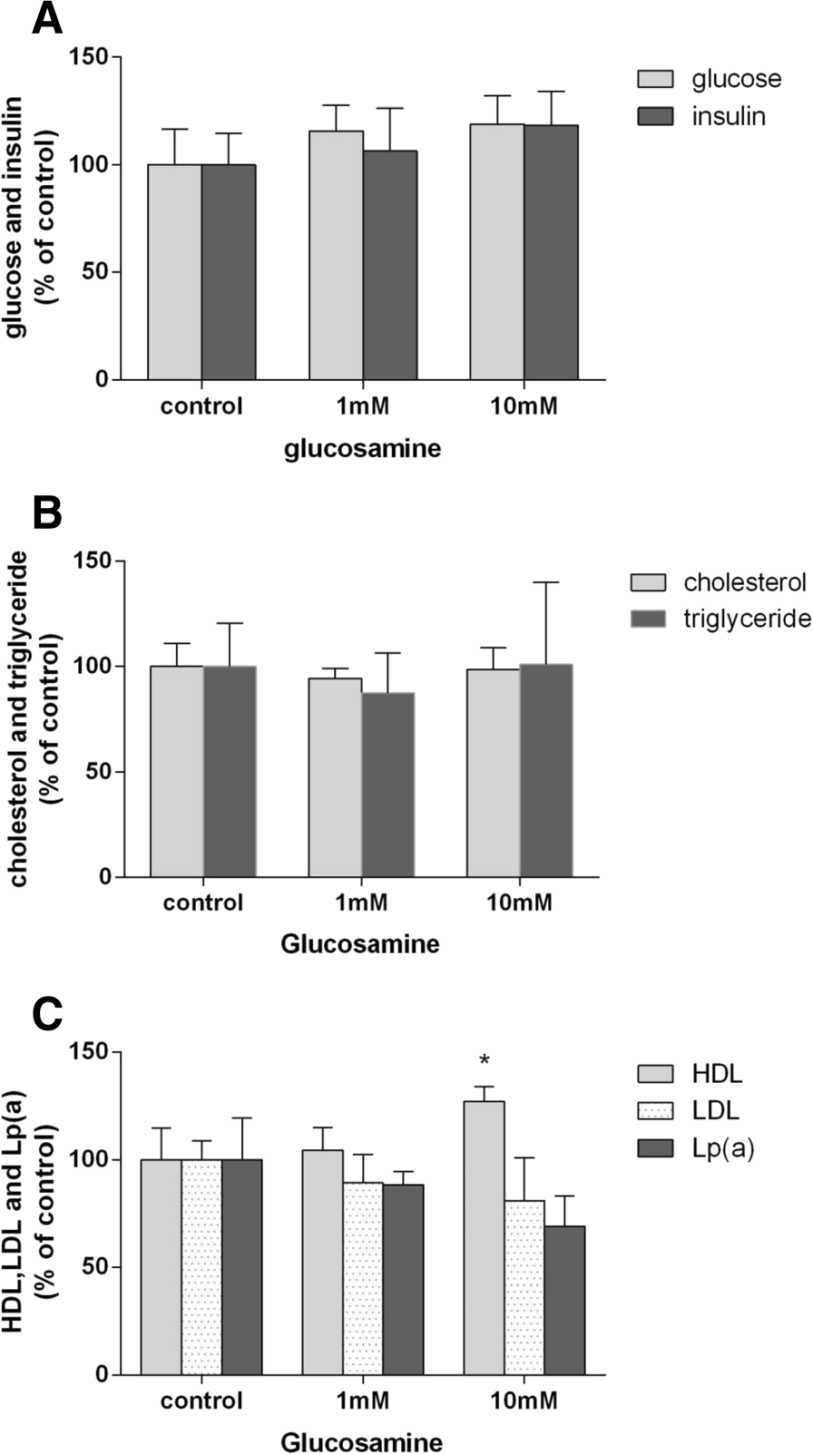
Effects of glucosamine on glucose, insulin, lipids and lipoproteins in rats. Serum glucose was measured by the glucose oxidase method. Insulin was measured by radioimmunoassay (Panel **a**). Total cholesterol, triglycerides (Panel **b**) and HDL-cholesterol, LDL-cholesterol and lipoprotein a (Panel **c**) were measured by standard clinical chemistry methods. The control group is set 100 %. *P <0.05 and **P < 0.01 vs. control

When rats were infused with saline (control group) or with glucosamine at 1 mmol/L or 10 mmol/L for 6 h, the mean concentration of serum glucosamine was about 0.04 mmol/L, 0.3 mmol/L, and 1.71 mmol/L respectively. The hepatic apoM mRNA levels were increased by 3.8 fold in rats infused with glucosamine at 1 mmol/L and by 8.6 fold when rats infused with glucosamine at 10 mmol/L compared to the rats infused with saline (Fig. 2a), while the serum apoM levels were not statistically different between these groups (Fig. 2b). As shown in Fig. 3a and b, there were no statistical significant changes on serum glucose, insulin, triglycerides and cholesterol levels when rats infused with wither glucosamine or saline. The serum HDLs were slightly increased by 4.6 % when rats infused with 1 mM glucosamine and increased by 27.7 % when rats infused with 10 mM glucosamine. The serum levels of LDL and lipoprotein a (Lpa) were slightly decreased in rats infused with 1 mM and 10 mM glucosamine compared to the control rats, but there was no statistical significance due to large individual variance (Fig. 3c).

## Discussion

ApoM may play an important role in HDL-mediated cholesterol reverse transport and is an attractive target for the treatment of diabetes, obesity, hyperinsulinemia and dyslipidemia [1, 2]. Hyperglycemia was shown to down-regulate the expression of apoM *in vivo* and *in vitro* [12], but the mechanism was still unknown. It has been reported that hyperglycemia could increase hexosamine biosynthesis pathway flux [15]. The hexosamine pathway constitutes a branch of the glycolytic pathway and is indispensable on regulation of glucose homeostasis [16]. Glucosamine is a prominent component of the hexosamine pathway and it has many physiological functions [17, 18]. The increased glucosamine via the hexosamine biosynthesis pathway impairs glucose transport and insulin action mediated by altered gene expression, and then changed the metabolism of glucose and lipoproteins [19]. We hypothesized that hyperglycemia induced down-regulation of apoM expression may mediate via the glucosamine pathway. Contrary to our hypothesis, the present results demonstrated an obvious up-regulative effect of glucosamine on apoM expression *in vivo* and *in vitro*.

In mammalian cells, under normal conditions, hexosamine pathway diverts 2–5 % of the fructose-6-phosphate derived from glucose into glucosamine-6-phosphate (GlcN-6-P), thus giving rise to obligatory substrates for the synthesis of glycoproteins and glycolipids [20]. Exogenous glucosamine is phosphorylated by hexokinases to GlucN-6-P after its entry into the cells. Endogenous GlucN-6-P is formed by fructose-6-P and glutamine by GFAT, which is the rate-limiting enzyme in the hexosamine pathway [21]. When the HepG2 cells were treated with azaserine, the inhibitor of GFAT, the endogenous hexosamine pathway was inhibited and the expression of apoM was suppressed significantly. When treated with glucosamine, the hexosamine pathway was continued by exogenous substrate and apoM mRNA was increased greatly. The results suggested both exogenous and endogenous hexosamine pathway being essential for the expression of apoM.

In the present study, there were no statistical significant changes on serum glucose and insulin levels after administration of glucosamine in rats. This may explain the different animal models used and short-term glucosamine infused in rats in the present study. It has been reported that the serum concentration of glucosamine is only about 0.0-0.04 mmol/L in the healthy people. After supplement of 30 g glucosamine intravenously, the serum concentration of glucosamine could be reach level of 1.42 mmol/L, however, it didn’t influence insulin sensitivity [22, 23]. In certain metabolic studies, after glucosamine administration orally or intravenously, could not demonstrate a significant change on blood concentrations of glucose or insulin [17, 24, 25].

Previous studies demonstrated that glucosamine could attenuate the vessel injury induced by hyperglycemia, promote apolipoprotein A1 expression and impair hepatic apolipoprotein B100 (apoB100) assembly and secretion [19, 26–28]. In this study, we confirmed that glucosamine enhanced apoM expression and both exogenous and endogenous glucosamine might be essential for the expression of apoM. It appeared that the increased intracellular content of glucosamine, a known mediator of insulin resistance, not be responsible for the reduced apoM expression in hyperglycemia condition. Moreover, the present study may open a new sight into the pathophysiological procedure of the apoM and apoM overexpression for a potential role on improving lipoprotein metabolism *in vivo*, which could be considered as a future therapeutic target against insulin resistance and type-2 diabetes.

## Conclusion

ApoM expression could be up-regulated by glucosamine *in vivo* and *in vitro*. Both exogenous and endogenous glucosamine are essential for the expression of apoM. Hyperglycemia induced down-regulation of apolipoprotein M expression is not via the hexosamine pathway.

## Materials and methods

### Materials

The established hepatoblastoma cell line, HepG2 cells, was from American Type Culture Collection (Manassas, VA, USA). Six-well cell culture clusters and 25-cm^2^ vented cell culture flasks were purchased from Gibco. Cell culture media were from the Gibco. Real-time RT-PCR reagents and control probe GAPDH andβ-actin were purchased from Shanghai Shenergy Biotech Co. Ltd. RevertAidTM First Strand cDNA Synthesis Kit was purchased from Fermenta. Glucosamine and azaserine were from Sigma. Rat fix-container was obtained from Beijing Xinhang Company (Beijing, China). Intravenous catheter was purchased from Becton Dickinson Medical Devices Co. Ltd. (Suzhou, China). Microinfusion pumps were purchased from Zhejiang University (Zhejiang, China).

### Cell cultures

HepG2 cells were cultured in 25-cm^2^ vented flasks containing DMEM with 10 % fetal calf serum (FCS) in the presence of benzyl-penicillin (100 U/ml) and streptomycin (100 μg/ml) under standard culture conditions (5 % CO_2_, 37 °C). Cells were seeded in six-well cell culture clusters, and were grown to 50–70 % confluence. Prior to experiment, cells were washed twice with phosphate buffered saline (PBS) and once with serum-free DMEM without antibiotic. Experimental medium contained DMEM with 0.5 % bovine serum albumin (BSA) and different concentrations of glucosamine and/or azaserine were added as specified in the legend to figures.

### Animal experiments

All the animal work was conducted in the compliance with the recommendations on the guide for the care and use of laboratory animals, and approved by the local ethic committee. Healthy adult male SD rats (body weight at 180–205 g) were obtained from Shanghai Slac Laboratory Animal Co. (Shanghai, China), and were kept in separated cages in a temperature-controlled (22 °C) room with 12-h light–dark cycle. Animals were provided with standard rodent chow and water ad libitum, and were acclimatized for 1 week before experiments. Rats were randomly divided into three groups five rats per group). All rats were deprived of food for 12 h before experiments. Animals were fixed on a special restraining-container and catheters were inserted into the tail vein. Rats were continuously infused (2.5 ml/h) with either 1 mmol/L glucosamine, 10 mmol/L glucosamine or 0.9 % saline (control group), respectively, by microinfusion pumps. After 6 h, rats were anesthetized and sacrificed. Blood samples were taken through inferior vena cava and the serum was separated and frozen at −70 °C. A specimen of liver tissue was sectioned and stored in liquid nitrogen.

### Measurement of plasma apoM levels

Serum apoM concentration was assayed by Western-blot analysis with a specific polyclonal rabbit anti recombinant human apoM antibody. In brief, serum was diluted with PBS buffer (1:20) and 20 μl diluted samples were fractionated by SDS–polyacrylamide gel electrophoresis (SDS–PAGE), transferred to nitrocellulose membrane, and incubated with rabbit anti recombination human apoM polyclonal antibodies. Horseradish peroxidase-conjugated swine anti-rabbit IgG were used as the secondary antibody. Bands corresponding to the apoM were visualized by an ECL + Plus Western blotting detection system (Amersham) . The relative amount of apoM was analyzed with Bio-Rad computer using Quantity One software.

### Total RNA extraction and real-time PCR

Total RNA of HepG2 cells and rat liver tissues were isolated by Trizol method, and sequentially reverted to cDNA according to the manufacturer’s instruction. Primer 5.0 Software was used to design rat and human apoM primers and probe used in the TaqMan assay. The rat and human apoM primers and probes were shown in Table 1. Relative standard curves for apoM, GAPDH and β-actin were performed to compensate the efficiency of PCR. A serial dilution of apoM cDNA was used to generate a standard curve by plotting the cycle threshold versus the log of input cDNA. Relative quantitative of mRNA was performed on Lightcycle. PCR of apoM was carried out in a 25ul reaction mixture containing 10 pmol of both forward and reverse primers, 10 pmol probe, 5 nmol dNTP, 2.5U Taq polymerase and 2ul cDNA. The mixture was preheated for 5 min to activate Taq polymerase. Then a 40-cycle two-step PCR was performed consisting of 5 s at 95 °C and 30 s at 60 °C. Real-time RT-PCR of GAPDH and β-actin was performed according to the manufacturer’s instruction. The threshold cycle (CT) is defined as fractional cycle number at which the reporter fluorescence reaches a certain level.

**Table 1:** Primers and fluorescent probes of human apoM, human GAPDH, rat apoM and ratβ-actin

Human apoM	
forward primer	5’ TGC CCC GGA AAT GGA TCT A 3’
reverse primer	5’ CAG GGC GGC CTT CAG TT 3’
probe	5’ FAM-CAC CTG ACT GAA GGG AGC ACA GAT CTC A-TAMRA 3’
Human GAPDH	
forward primer	5’GGA AGG TGA AGG TCG GAG TC 3’
reverse prime	5’CGT TCT CAG CCT TGA CGG T 3’
probe	5’FAM- TTT GGT CGT ATT GGG CGC CTG -TAMRA3’
Rat apoM	
forward primer	5’ ACAAAGAGACCCCAGAGCCC 3’
reverse primer	5’ TCCATGGTGGGAGCCG 3’
probe	5’ FAM- ACCTGGGCCTGTGGTACTTTATTGCTGG -TAMRA 3’
Rat β-actin	
forward primer	5’ GCCACTGCCGCATCCTCT 3’
reverse prime	5’ CTGGAAGAGAGCCTCGGGG 3’
probe	5’FAM-AGCTGCCTGACGGTCAGGTCATCACTATC -TAMRA3’

### Chemical measurement

Blood glucose was measured by the glucose oxidase method. Insulin was measured by radioimmunoassay (Beijing Atom Hightech Co, China). Serum glucosamine, total cholesterol, triglycerides, HDL-cholesterol and LDL-cholesterol were measured by standard clinical chemistry methods.

### Statistics

Statistical analyses were performed with the Prism on an Apple computer. Comparisons among groups were statistically analyzed by one-way ANOVA followed by unpaired Student’s *t*-test or analyzed by two-tailed Mann–Whitney *U* test. A *P* value less than 0.05 was considered as statistically significant.

## Abbreviations

apoM: Apolipoprotein M
apoA1: Apolipoprotein A1
CHD: Coronary heart disease
SNP: Single nucleotide polymorphism
IGF-I: Insulin-like growth factor I
IGF-IPP: IGF-I potential peptide
GFAT: Glutamine:fructose-6-phosphate amidotransferase
